# Trypsin-protease activated receptor-2 signaling contributes to pancreatic cancer pain

**DOI:** 10.18632/oncotarget.18696

**Published:** 2017-06-27

**Authors:** Jiao Zhu, Xue-Rong Miao, Kun-Ming Tao, Hai Zhu, Zhi-Yun Liu, Da-Wei Yu, Qian-Bo Chen, Hai-Bo Qiu, Zhi-Jie Lu

**Affiliations:** ^1^ Department of Anesthesiology and Intensive Care Medicine, Eastern Hepatobiliary Surgery Hospital, Second Military Medical University, Shanghai 200433, China; ^2^ Department of Anesthesiology, Maternity and Infant Health Hospital of Putuo District, Shanghai 200062, China; ^3^ Department of Anesthesiology, No.101 hospital of PLA, Wuxi 214000, China

**Keywords:** pancreatic cancer, pain, trypsin, protease activated receptor-2, pancreatic cancer pain model

## Abstract

Pain treatment is a critical aspect of pancreatic cancer patient clinical care. This study investigated the role of trypsin-protease activated receptor-2 (PAR-2) in pancreatic cancer pain. Pancreatic tissue samples were collected from pancreatic cancer (n=22) and control patients (n=22). Immunofluorescence analyses confirmed colocalization of PAR-2 and neuronal markers in pancreatic cancer tissues. Trypsin levels and protease activities were higher in pancreatic cancer tissue specimens than in the controls. Supernatants from cultured human pancreatic cancer tissues (PC supernatants) induced substance P and calcitonin gene-related peptide release in dorsal root ganglia (DRG) neurons, and FS-NH_2_, a selective PAR-2 antagonist, inhibited this effect. A BALB/c nude mouse orthotopic tumor model was used to confirm the role of PAR-2 signaling in pancreatic cancer visceral pain, and male Sprague-Dawley rats were used to assess ambulatory pain. FS-NH_2_ treatment decreased hunch scores, mechanical hyperalgesia, and visceromotor reflex responses in tumor-bearing mice. In rats, subcutaneous injection of PC supernatant induced pain behavior, which was alleviated by treatment with FS-NH_2_ or FUT-175, a broad-spectrum serine protease inhibitor. Our findings suggest that trypsin-PAR-2 signaling contributes to pancreatic cancer pain *in vivo*. Treatment strategies targeting PAR-2 or its downstream signaling molecules might effectively relieve pancreatic cancer pain.

## INTRODUCTION

Pain treatment in pancreatic cancer patients is one of the most critical aspects of clinical care [1]. Possible causes of pancreatic cancer pain include infiltration of nerve sheaths and neural ganglia, increased ductal and interstitial pressure, and gland inflammation [2, 3]. However, pancreatic cancer pain mechanisms are not fully understood, and cancer pain assessment and management strategies are currently inadequate.

Protease-activated receptor-2 (PAR-2) belongs to a subfamily of four protease-activated G-protein-coupled receptors [4]. PAR-2 is activated in many cell types by trypsins and mast cell tryptase, and participates in a variety of physiological and pathophysiological responses, including gastrointestinal smooth muscle relaxation, cancer cell migration, neurogenic inflammation, and nociceptive signaling [5]. Injecting PAR-2 agonist into the joints, paws, or subarachnoid space in animal models reportedly elicits acute inflammatory hyperalgesia through sensitization of pain-sensing primary sensory neurons [6, 7]. PAR-2 also plays a critical role in irritable bowel syndrome (IBS)- and pancreatitis-related visceral pain. Released proteases can directly activate sensory neurons and generate hypersensitivity symptoms through PAR-2 activation in IBS [8–10].

In the pancreas, PAR-2 is expressed on pancreatic acinar and ductal cell luminal surfaces, and in pancreatic sensory nerves [11]. Trypsin infusion into the pancreatic duct activates pancreas-specific afferent neurons *in vivo* and increases spinal FOS expression dose-dependently [12]. Trypsin is also an endogenous key mediator of pancreatitis. Premature cleavage of trypsinogen in pancreatic acinar cells releases the activated serine protease, trypsin, resulting in cellular damage and inflammatory cell infiltration [13, 14]. Trypsin may activate PAR-2, which localizes to pancreatic sensory nerve fibers and promotes dorsal root ganglia (DRG) sensory neuron nociceptive sensitization in pancreatic cancer patients [15]. In this study, we used human pancreatic cancer tissue samples, DRG neurons, and a pancreatic cancer pain model to explore the roles of trypsin and the PAR-2 pathway in pancreatic cancer pain.

## RESULTS

### PAR-2 and PGP9.5 colocalized in pancreatic cancer specimens

**Figure 1:**
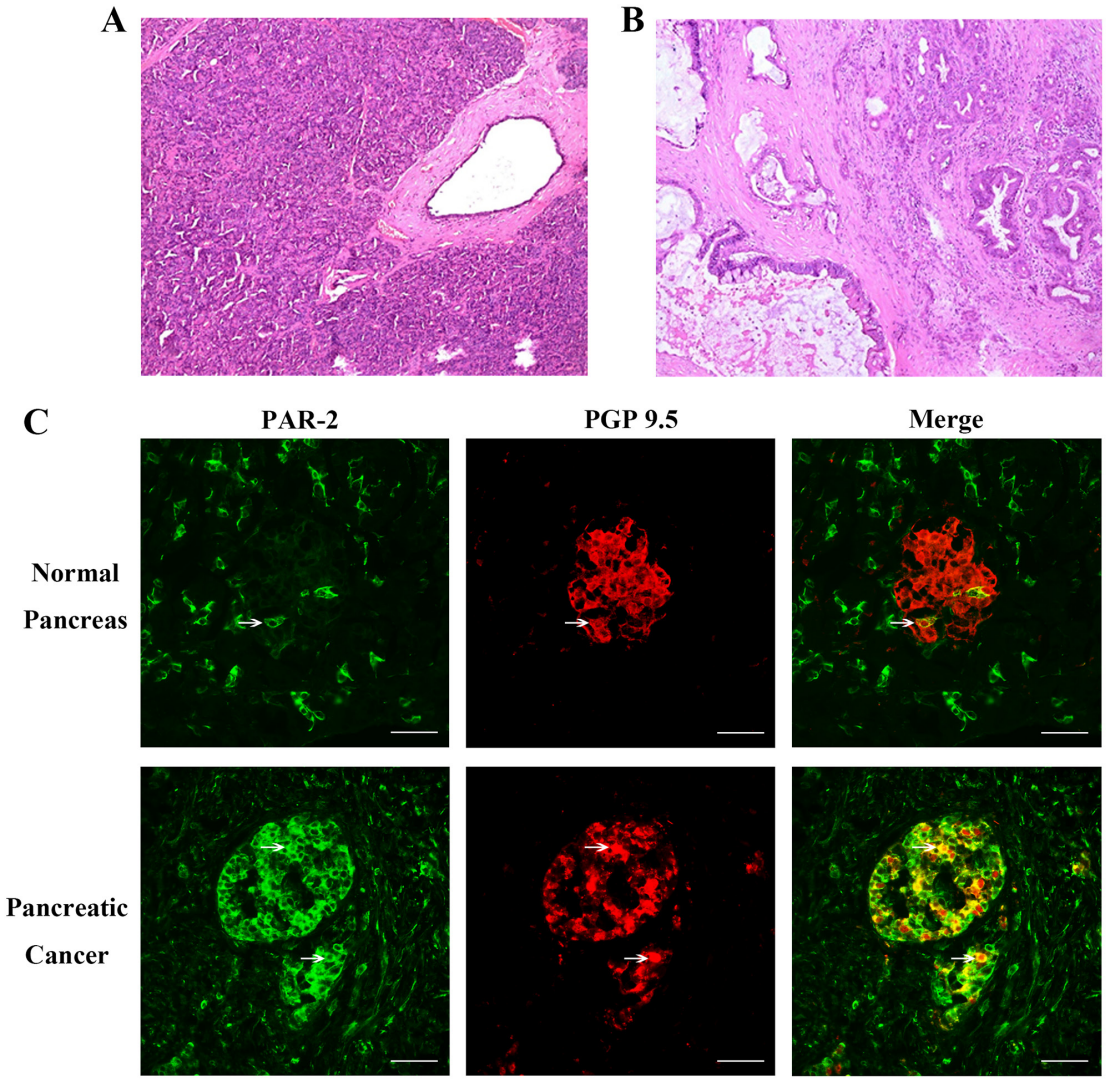
Histology of pancreatic tissue specimens and immunofluorescence assay for PAR-2 and PGP 9.5 in human pancreatic tissue specimens Hematoxylin and eosin stained section of pancreatic tissues from control patients **(A)** and patients with pancreatic cancer **(B)**. Scale bar = 50μm. **(C)** To visualize neuronal axons, tissues were immunostained with a monoclonal antibody to PGP-9.5. The colocalization of PAR-2 and PGP 9.5 was significant increased in specimen from pancreatic pancreatic cancer tissue. White arrow indicated the PAR-2 and PGP 9.5 co-expressing cells. Scale bar = 50μm.

Destruction of normal pancreatic tissue structure was confirmed in human pancreatic cancer specimens (Figure 1B). Immunofluorescence (IF) analyses showed that while PAR-2 was expressed in epithelial cells and the cellular matrix from both pancreatic cancer and control specimens, PAR-2 levels were increased in pancreatic cancer (Figure 1C). PAR-2 and the neuronal marker, protein gene product-9.5 (PGP9.5), colocalized in pancreatic cancer specimens (Figure 1C). We also did immunochemistry study for PAR-2 and PGP 9.5 expression in human pancreatic tissues, and the statinning patterns of PAR-2 and PGP 9.5 immuno-active signals are the same as which had been presented in the immunofluorescence study (Supplementary Figure 1).

### Trypsin level and protease activity were higher in PC supernatants than control tissues

**Figure 2:**
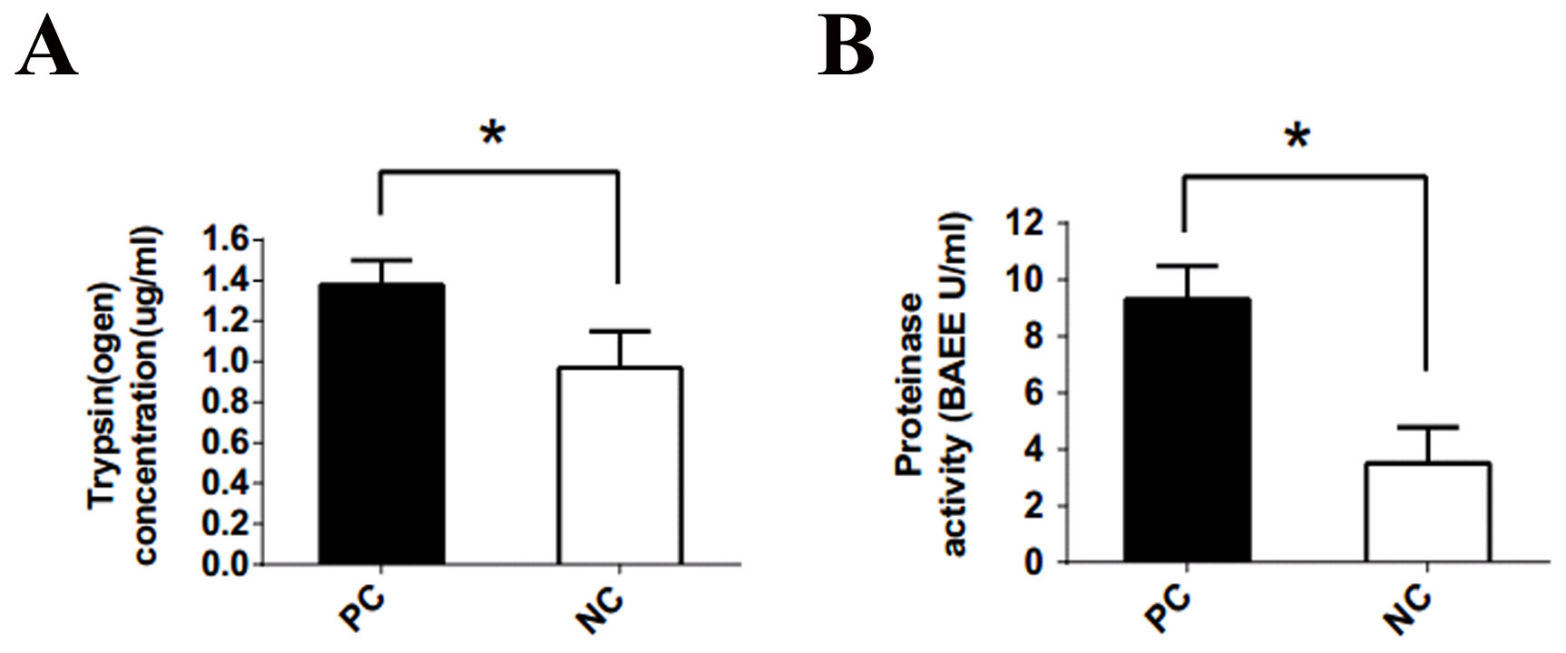
Trypsin(ogen) concentration and protease activity released from pancreatic cancer and control specimens **(A)** Trypsin (ogen) released from the specimens of pancreatic cancer tissue was significantly higher than that released from the non-tumor specimens (1.38±0.12 vs 0.97±0.18 μg/ml). **(B)** Trypsin activity determined by the substrate BAEE. Protease activity released from specimens of pancreatic cancer patients was 2 to 3 fold higher than the activity in control specimens (9.32±1.17 vs 3.50±1.28 BAEE U/ml). PC: supernatant cultured from pancreatic cancer tissues. NC: supernatant cultured from normal pancreatic tissues. *P<0.05; n = 22 in each group.

Visual analog scale (VAS) scores of patient abdominal pain differed between pancreatic cancer and control patients, while age, sex and weight were similar (Table 1). Higher trypsin levels were observed in pancreatic cancer (PC) supernatants (supernatants from cultured human pancreatic cancer tissues) than in normal control (NC) supernatants (supernatants from cultured human normal pancreatic tissues) (Figure 2A). Since trypsin is a pancreatic enzyme with protease activity, we used its substrate, BAEE (Na-benzoyl-L-arginine ethyl ester), to assess protease activity. Protease activity in pancreatic cancer tissues was 2–3-fold higher than in control tissues (Figure 2B).

**Table 1:** Clinical data of included patients

Characteristic	Control (n=22)	Pancreatic cancer (n=22)
Age (yr, range)	49-74	29-74
Sex (no., F/M)	10/12	6/16
Weight (kg, range)	50-77	46-70
VAS†	1.1±0.4	5.9±0.8

† *P* < 0.05

### PC supernatant induced PAR-2-dependent somatic nociception *in vivo*

**Figure 3:**
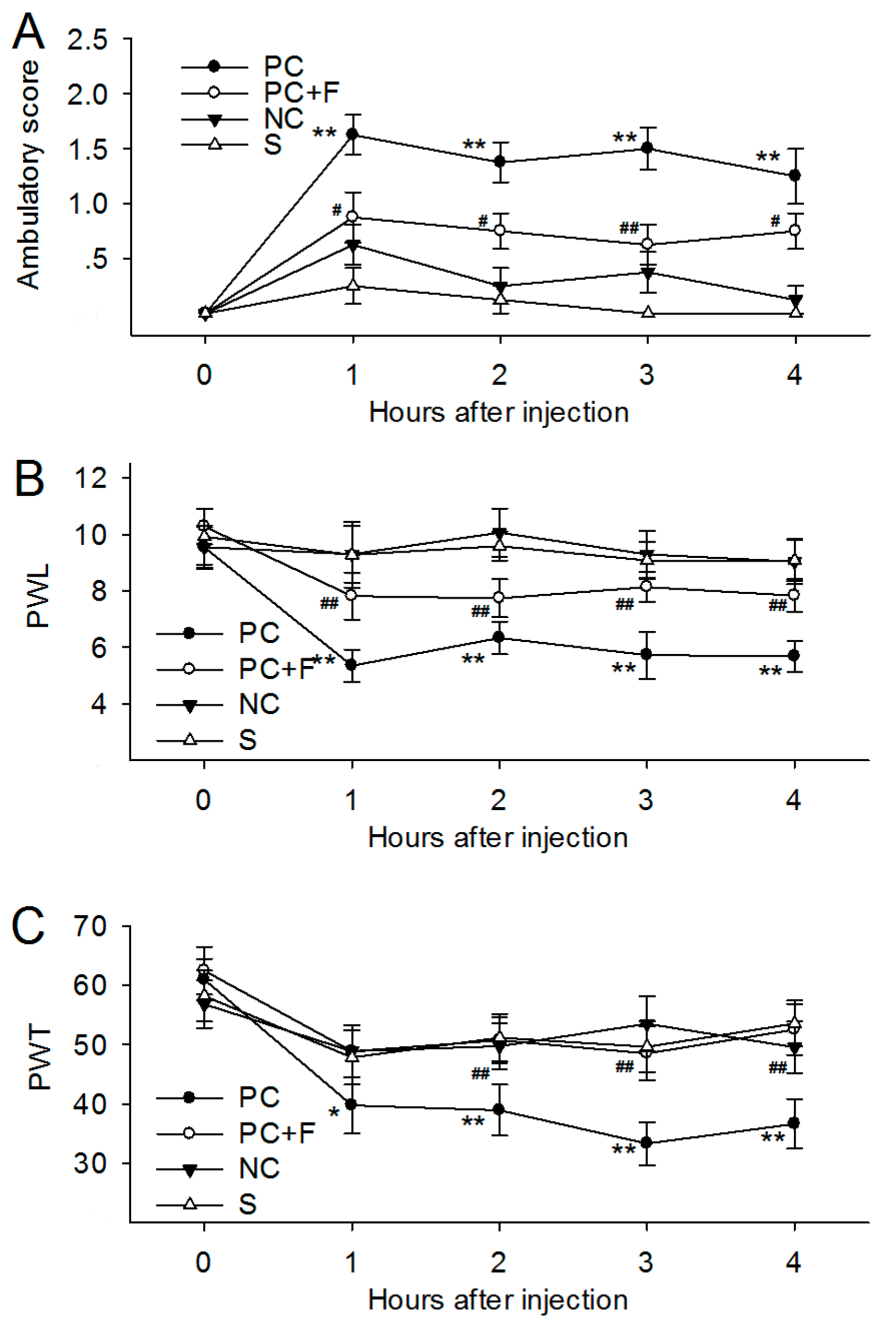
Intraplantar administration with supernatants cultured from pancreatic cancer tissues (PC supernatant, 25ul) induced ambulatory pain, thermal and mechanical hyperalgesia **(A)** Rats receiving injection of PC supernatant exhibited higher ambulatory score in 4h after injection. Addition of FUT-175 (50 μg/ml) into the PC supernatants immediately before injection reduced ambulatory pain in rats at each time point during observation. **(B)** Rats receiving injection of PC supernatant exhibited decreased PWL in response to thermal stimulus. **(C)** Rats receiving injection of PC supernatants exhibited decreased PWT in response to mechanical stimulus. Addition of FUT-175 reversed the PWL and PWT induced by PC supernatants. PC: supernatants cultured from pancreatic cancer tissues. NC: supernatants cultured from normal pancreatic tissues. F: a broad-spectrum serine protease inhibitor, FUT-175. S: sham control. *P<0.05 compared with normal group; **P<0.01compared with normal group; # P<0.05 compared with tumor group; ## P<0.01 compared with tumor group. n=6 in each group.

To determine whether PC supernatant affected pain-related behaviors, we measured ambulatory-evoked pain scores. Ambulatory pain was not observed in rats that received NC supernatant intraplantar injections. Rats injected with PC supernatant exhibited an apparent limp on the injected hind limb following injection. Decreased injected limb use was observed 4 h after injection (Figure 3A). Administration of PC supernatant with FUT-175 (50 μg/ml), a broad-spectrum serine protease inhibitor, reduced ambulatory pain in rats (Figure 3A).

Intraplantar administration of PC supernatant in rats decreased paw withdrawal latency (PWL) in response to thermal stimulus, while NC supernatant had no effect during the 4 h of observation time (Figure 3B). Similarly, injecting PC supernatant, but not NC supernatant, into the rat paw acutely decreased paw withdrawal threshold (PWT) in response to mechanical stimulation, suggesting the development of mechanical hyperalgesia (Figure 3C). PC supernatant-induced PWL and PWT scores decreased with FUT-175 administration. These results indicated that protease activity in PC supernatants induced ambulatory pain and thermal and mechanical hyperalgesia.

**Figure 4:**
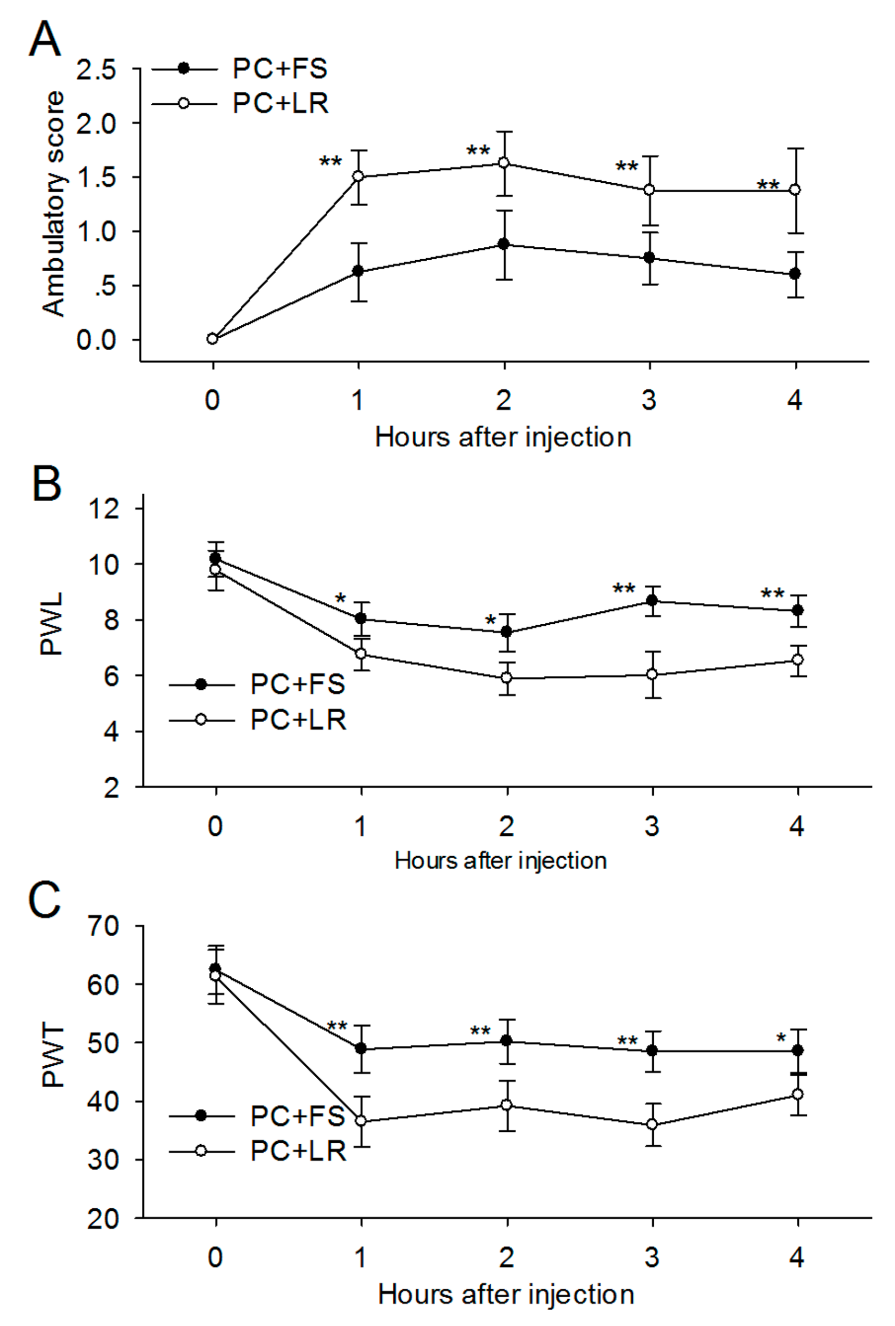
PAR-2 antagonist reversed the ambulatory pain, thermal and mechanical hyperalgesia induced by PC supernatant **(A)** Addition of PAR-2 antagonist FS-NH_2_ (100 μM) into the PC supernatant (25μl) immediately before injection reduced ambulatory pain in rats at each time point during observation. **(B)** FS-NH_2_ increased the PWL in rats received PC supernatants injection in response to thermal stimulus. **(C)** FS-NH_2_ increased the PWT in rats received PC supernatants injection in response to mechanical stimulus. PC: supernatants cultured from pancreatic cancer tissues; FS: FSLLRY-NH_2_, PAR-2 antagonist peptide; LR: LRGILS-NH_2,_ control peptide. *P<0.05, **P<0.01 compared with control peptide group; n=6 in each group.

Intraplantar injection of PC supernatant with FS-NH_2_ (100 μM), a selective PAR-2 antagonist peptide, decreased ambulatory pain scores in rats (Figure 4A) during observation. FS-NH_2_ also decreased PWL and PWT (Figure 4B–4C) in the injected hind limb during observation compared to the control peptide, LR-NH_2_ (100 μM). These results indicated that PAR-2 mediates PC supernatant-induced somatic nociception.

### PAR-2 participated in PC supernatant-induced primary sensory neuron activation

**Figure 5:**
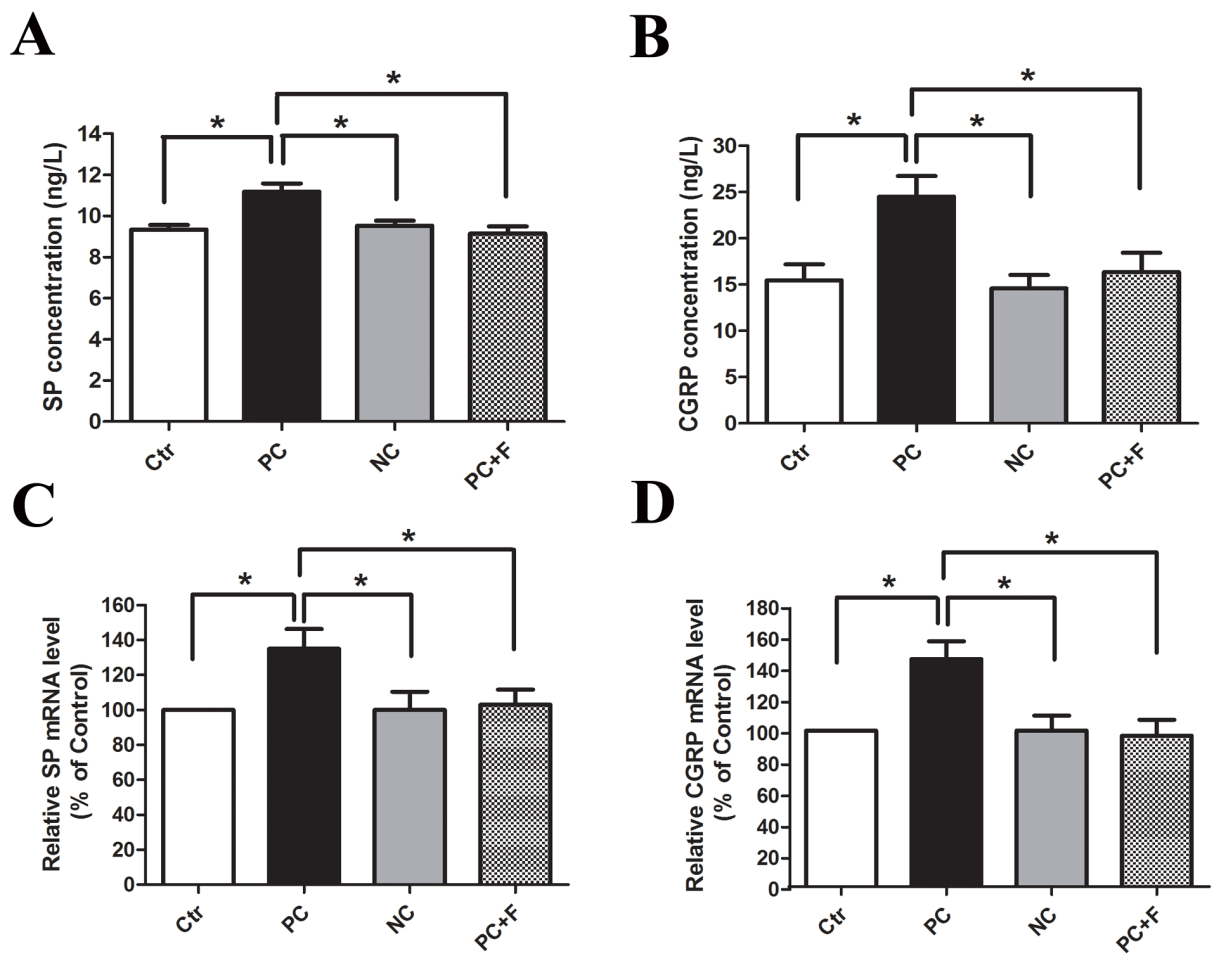
PC supernatant induced DRG neurons releasing of SP and CGRP in a protease-dependent way After incubation for 2h, the releasing of SP **(A)** and CGRP **(B)** form cultured DRG neurons were higher in PC supernatant group than that in NC supernatant group. Similarly, the mRNA levels for SP **(C)** and CGRP **(D)** were significantly increased in DRG neurons treated with PC supernatants. Co-incubation with protease inhibitor FUT-175 (50μg/ml) and PC supernatant reduced this neuropeptide release effect in cultured DRG neurons. Ctr: unstimulated DRG. PC: supernatants cultured from pancreatic cancer tissues. NC: supernatants cultured from normal pancreatic tissues. F: a broad-spectrum serine protease inhibitor, FUT-175.*P<0.05 n=6 in each group.

**Figure 6:**
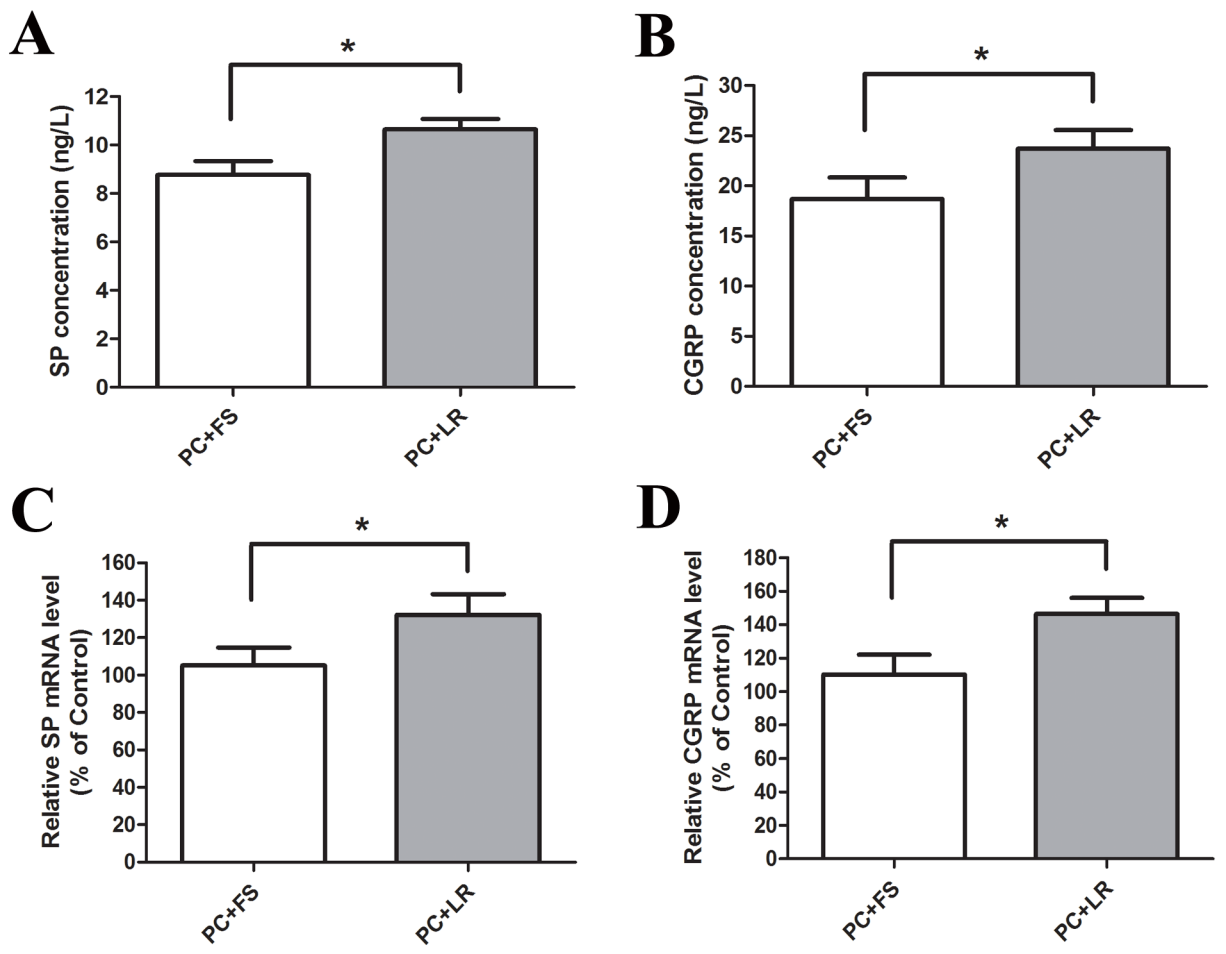
PAR-2 antagonist reversed PC supernatant induced releasing of SP and CGRP in cultured DRG neurons **(A and B)** FS-NH_2_ (100μM), a selective antagonist peptide of PAR-2, caused a marked decrease in SP and CGRP releasing in the medium of cultured DRG neurons in contrast to the control peptide LR-NH_2_ (100μM). **(C and D)** Similarly, FS-NH_2_ decreased the SP and CGRP mRNA levels in DRG neurons. FS: FSLLRY-NH_2_, PAR-2 antagonist peptide; LR: LRGILS-NH_2,_ control peptide. *P<0.05 compared with control peptide group; n=6 in each group.

Evidence suggests links between nociception and calcitonin gene-related peptide (CGRP) or substance P (SP) in the primary afferent pathway [16]. Incubation of cultured DRG neurons with PC supernatant upregulated SP and CGRP expression (Figure 5C–5D) and release (Figure 5A–5B) compared to NC supernatant. PC supernatant-treated DRG neurons that were pre-incubated with FUT-175 (50 μg/ml) exhibited reduced SP and CGRP expression (Figure 5C–5D) and release (Figure 5A–5B) into culture medium, suggesting that PC supernatant-induced activation of primary sensory neurons is protease-dependent. We then incubated DRG neurons together with PC supernatant and either FS-NH_2_ (100 μM) or the control peptide, LR-NH_2_ (100 μM). FS-NH_2_ again decreased SP and CGRP expression (Figure 6C–6D) and release (Figure 6A–6B) into culture medium. These results suggested that PC supernatant activated sensory neurons through PAR-2.

### FS-NH_2_ alleviated pancreatic cancer pain in naïve mice

**Figure 7:**
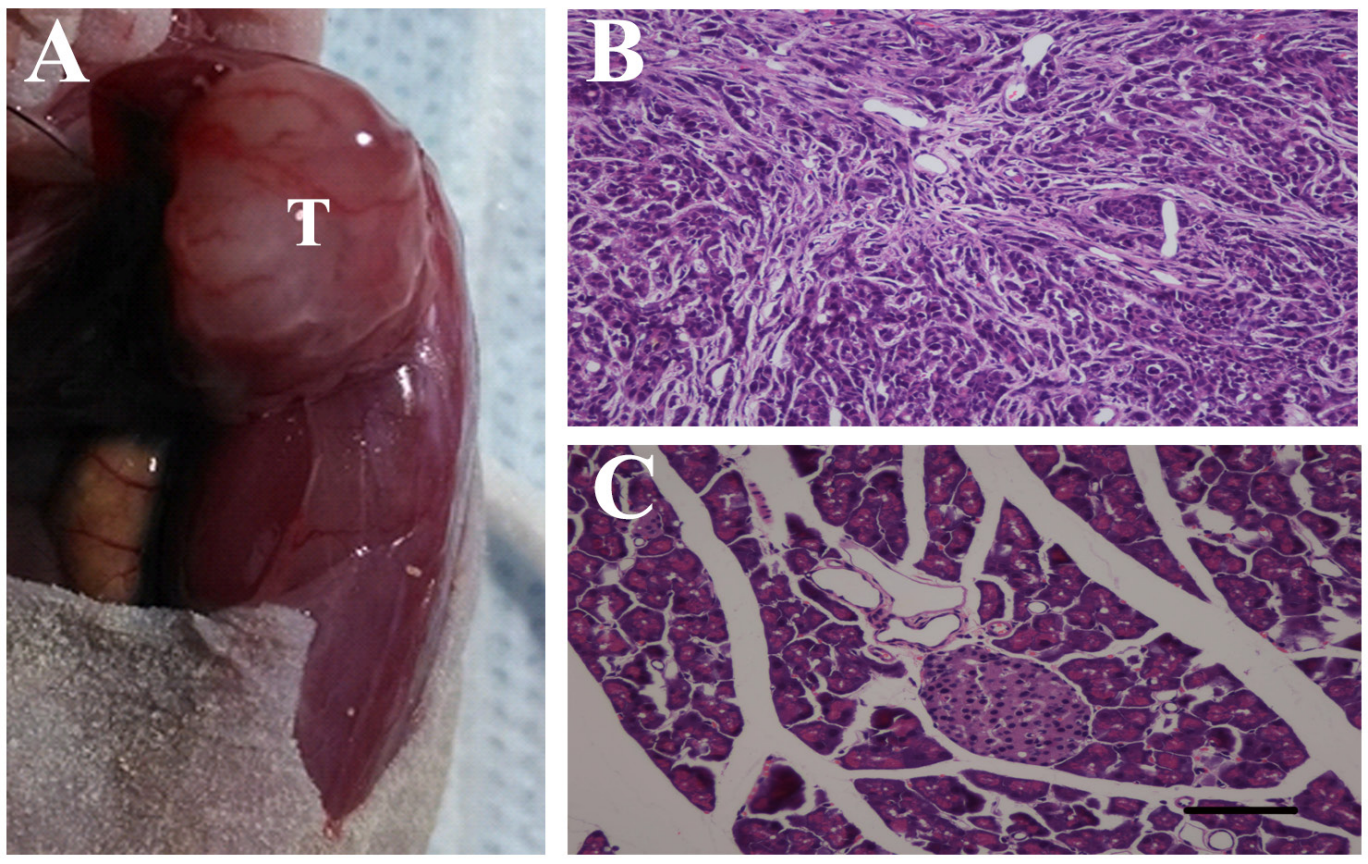
Pancreatic cancer model build by human pancreatic cancer cell line SW1990 in male BALB/c nude mice **(A)** SW1990 cells (5×10^6^) in a volume of 20 μL were injected into the body of the pancreas, after 3 weeks, tumors were apparent on the pancreas (T). **(B)** Immunohistochemistry examination confirmed the success planting of SW1990 cells on the pancreas of naïve mice. **(C)** Hematoxylin and eosin stained section of pancreatic tissues from control mice. Scale bar = 50μm.

**Figure 8:**
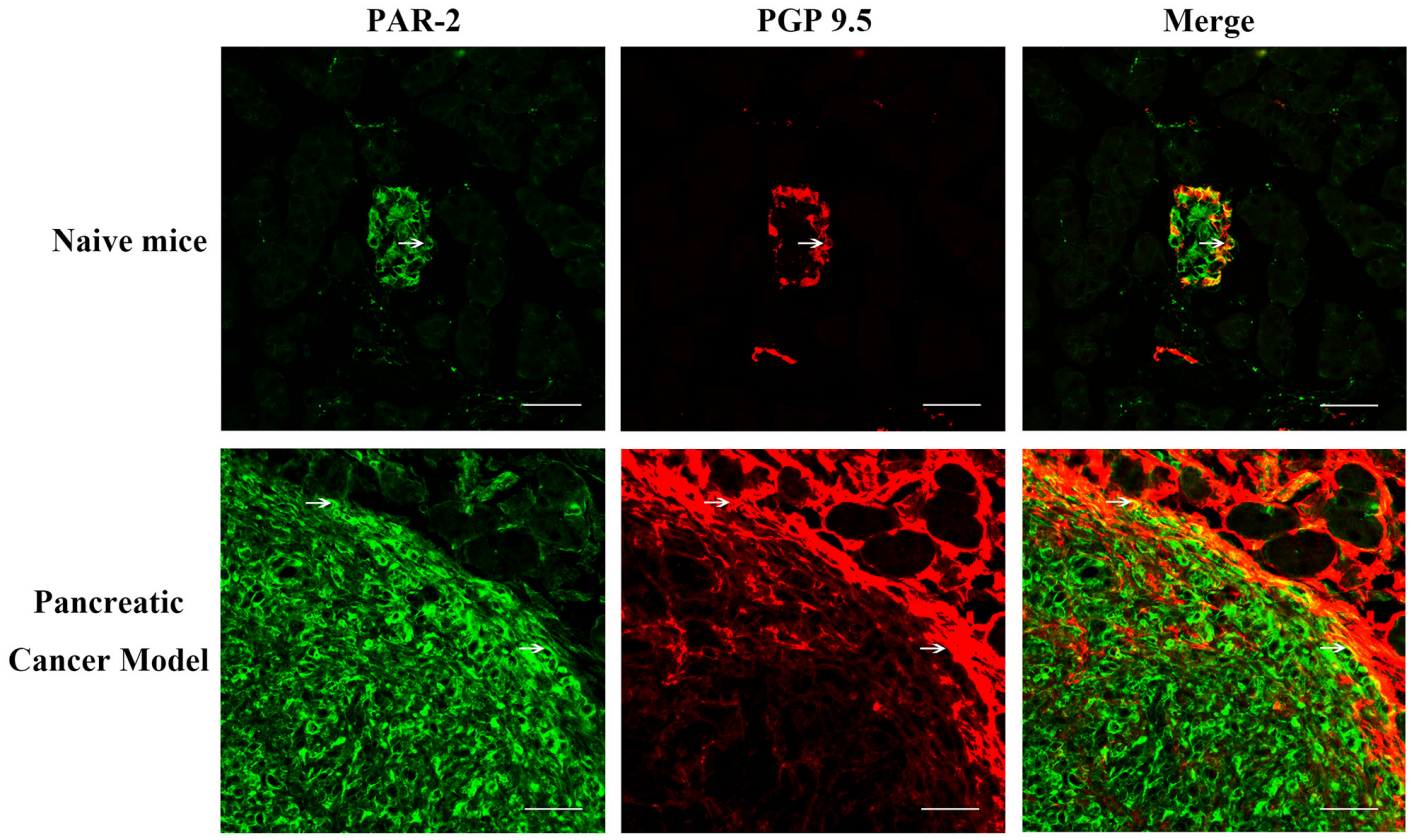
Immunofluorescence assay for PAR-2 and PGP 9.5 in pancreatic tissue specimens from naïve mice Pancreatic cancer tissues of naïve mice were immunostained with antibody to PGP-9.5 and PAR-2. PAR-2 is expressed in the implanted SW1999 orthotropic pancreatic tumor, while PGP 9.5 immuno-active signals scattered cycling the tumor area in pancreatic cancer model. White arrow indicated the PAR-2 and PGP 9.5 co-expressing cells. Scale bar = 50μm.

**Figure 9:**
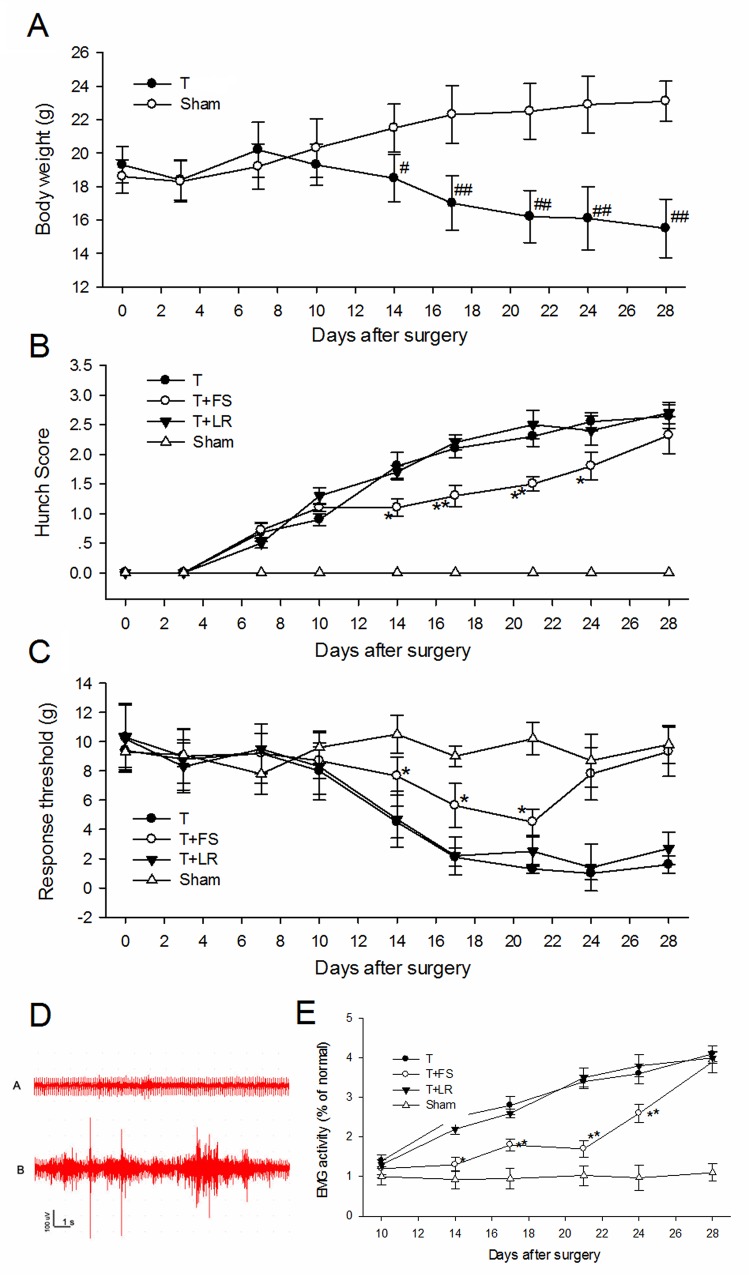
PAR-2 antagonist alleviated pancreatic cancer pain in nude mice **(A)** Body weights of mice that received SW1990 cell or sham injection. From 2 weeks to 4 weeks, tumor-bearing mice weighed significantly less than sham mice. **(B)** Hunching behavior of mices with injection of SW1990 cell and PAR-2 antagonist peptide. A significant increase in hunching behavior score in tumor-bearing mice group was observed from day 14 after surgery. Intraperitoneal injection of PAR-2 antagonist peptide FS-NH_2_ (500μl, 10mM) every day from 14 to 21 days decreased the hunching behavior score in tumor-bearing mice. **(C)** Mice withdrawal response to mechanical stimulation with electric von Frey hairs in the abdomen. PAR-2 antagonist peptide FS-NH_2_ (500μl, 10mM), but not control peptide LS-NH_2_ (500μl, 10mM), significantly inhibited the mechanical hyperalgesia in abdomen. **(D)** Representative EMG graphs of acromiotrapezius muscle of tumor-bearing mice (lower) and sham mice (upper). **(E)** PAR-2 antagonist peptide FS-NH_2_ (500μl, 10mM), but not control peptide LS-NH_2_ (500μl, 10mM), significantly inhibited the EMG activity of acromiotrapezius muscle in tumor-bearing mice. T: tumor-bearing mice; FS: FSLLRY-NH_2_, PAR-2 antagonist peptide; LR: LRGILS-NH_2,_ control peptide. # P<0.05 compared with sham group; ## P<0.01 compared with sham group; * P<0.05 compared with control peptide group; **P<0.01 compared with control peptide group. n=6 in each group.

We studied the effects of PAR-2 antagonism on pancreatic cancer pain in a naïve mouse pancreatic cancer pain model. Tumors were apparent on the pancreas three weeks post- SW1990 cell injection (Figure 7A), as confirmed by immunohistochemical examination (Figure 7B). IF analyses showed PAR-2 and PGP9.5 colocalization in mouse pancreatic cancer tissues (Figure 8). Body weights were recorded for mice that received SW1990 cell or sham injections after surgery (Figure 9A).

Treated mice were assessed every week post-surgery for hunching behavior consistent with visceral pain [7]. While there was no evidence of hunching behavior in animals that received sham injections, increased hunching behavior scores were observed from day 14 post-surgery in tumor-bearing mice (Figure 9B).

We measured withdrawal responses to mechanical stimulation with electric von Frey hairs in the abdomen. Sham-injected mice showed no differences from baseline responses after surgery (Figure 9C). However, SW1990 cell-injected mice showed intensely referred mechanical hyperalgesia in the abdomen 14–28 days after surgery.

Visceral hyperalgesia produces a visceromotor reflex (VMR), which is quantified by electromyographic (EMG) recordings from various external muscles in response to noxious visceral stimuli. Injection of SW1990 cells into the mouse pancreas caused a visible pain response with increased EMG activity of the acromiotrapezius muscle compared with sham-injected mice (Figure 9D). EMG activity remained unchanged from baseline in sham mice during the observation period (Figure 9E). EMG activity (as measured by AUC) increased 193% over the average baseline in mice injected with SW1990 cells (Figure 9E) 14 d post-surgery, and increased progressively from 14–28 d post-surgery. These results indicated that pain behavior progressed in SW1990 cell tumor-bearing mice.

Since FS-NH_2_ inhibited PC supernatant-induced activation of primary sensory neurons and somatic nociception, we evaluated its contribution to pancreatic cancer pain. We assessed the effects of daily intraperitoneal FS-NH_2_ administration (500 μl, 10 mM) from 14 to 21 d after SW1990 cell injection. LS-NH_2_ (500 μl, 10 mM) was used as a control. FS-NH_2_, but not LS-NH_2_ treatment immediately decreased hunching behavior score (Figure 9B). Hunching behavior returned 7 d after the last FS-NH_2_ injection. Similarly, FS-NH_2_, but not LS-NH_2_ inhibited mechanical hyperalgesia in the abdomen (Figure 9C) and acromiotrapezius muscle EMG activity (Figure 9E) in tumor-bearing mice. The FS-NH_2_ anti-nociception effect lasted 3 d and returned 7 d after the last intraperitoneal injection. These results confirm that PAR-2 mediates pancreatic cancer pain.

## DISCUSSION

This study found that trypsin levels and protease activities were increased in human pancreatic cancer specimens compared to normal pancreas. Supernatants from cultured tumor tissue specimens activated DRG neurons *in vitro* and generated somatic nociception *in vivo*, which was effectively inhibited by the PAR-2 antagonist, FS-NH_2_, indicating that these effects were PAR-2 dependent. Intraperitoneal FS-NH_2_ administration also inhibited pain-related behaviors in pancreatic tumor-bearing mice, suggesting that PAR-2 might be an effective therapeutic target for reducing pancreatic cancer pain.

Trypsin triggers nociceptive responses in the pancreas [12, 17]. When persistent noxious stimulation leads to sensitization, small changes in pressure or inflammatory mediator levels may activate sensitized pancreatic nociceptive neurons, leading to amplified pain response [18]. Elevated endogenous trypsin levels and protease activity in pancreatic cancer tissues may have two sources. First, pancreatic tumor cells can secrete tryptase-like molecules, and the tumor microenvironment contains a variety of proteases that can activate PAR-2, which regulates tumor cell proliferation, differentiation, and invasion [19]. Second, pancreatic tumor growth and invasion destroys the integrity of the pancreatic duct and parenchyma, resulting in further trypsin release and activation. We observed that trypsin in normal pancreatic tissues did not induce SP and CGRP release *in vitro* and elicited no pain response *in vivo*. This may be due to PAR-2 dose-dependent activation, as previous findings showed that trypsin infusion into the pancreatic duct should reach the subinflammatory concentrations necessary to induce both behavioral nociceptive responses and spinal neuron activation [12].

We used a PGP9.5-specific antibody to visualize neuron axons in human pancreatic tissues. PGP9.5, expressed in nerves and some neuroendocrine cells, is a neuron-specific peptide [20] used as a marker for sensory nerve fibers, including small-diameter fibers transmitting pain and large fibers transmitting proprioception [21]. Tomita, *et al.* visualized PGP9.5-positive fine nerve fibers in normal pancreas in intra- and inter-islet, inter-acinar and perivascular stroma, along with scattered ganglion cells [20]. Similarly, we detected immunostained fibers scattered around the periductal, peri-islet, and inter-islet areas. PAR-2 and PGP 9.5 colocalization was previously observed in rodent trigeminal sensory neurons [22]. We also observed that PGP9.5-positive nerve fibers innervating the pancreas co-express PAR-2, especially in pancreatic cancer patients. Modified nerve morphology and disturbed intrapancreatic nerve homeostasis are likely origins of pancreatic cancer pain [23]. We hypothesized that elevated trypsin levels can activate PAR-2 on intrapancreatic nerve endings to generate and maintain pancreatic cancer pain.

The trypsin-PAR-2 pathway reportedly mediates acute visceral pain in pancreatitis [24, 25]. We found that tumor tissue supernatants activated sensory neurons and decreased pain-related behavior thresholds, suggesting that trypsin-PAR-2 signaling may be involved in pancreatic cancer visceral pain induction and maintenance *in vivo*. PAR-2 plays a critical role in peripheral nociception and visceral hyperalgesia [26], and PAR-2 activation in trigeminal nociceptive sensory neurons leads to pain transmission. PAR-2 activation in the colon and jejunum initiates hyperalgesic responses and trans-activation of downstream pain receptors in spinal cord lamina I and II [27, 28]. A previous study reported that administration of supernatant from human carcinoma cells into mice hind paws caused mechanical allodynia, but this effect was absent in PAR-2-deficient mice [17]. Our study employed supernatants from cultured human pancreatic cancer tissues, rather than cell lines, and demonstrated a role for PAR-2 in sensory neuron activation and pancreatic cancer-induced pain. We also found that FS-NH_2_ administration reversed nociceptive behaviors in a mouse pancreatic cancer pain model.

Thermal hyperalgesia in chronic pancreatitis is associated with PAR-2 upregulation in DRGs [29]. Downstream of PAR-2, CGRP and SP, both considered pain neurotransmitters, were observed in nerve fibers from chronic pancreatitis patients. PAR-2 activation can lead to SP and CRRP release from afferent C-fibers in a Ca^2+^-dependent process. We observed that PC supernatant-induced DRG neurons released SP and CGRP in a PAR-2-dependent fashion. While it is possible that SP and CGRP directly activate primary spinal afferent neurons, PAR-2 activation might induce local neurotransmitter release from peri-pancreatic nerve fibers, triggering neurogenic inflammation events, activated immune cell recruitment, and pain. As the increased proteinase activity observed in our study is also associated with pancreatic inflammation, PAR-2 activation likely plays a role in the generation of pancreatic cancer pain associated with neuron inflammatory states. However, previous work using a pancreatic duct infusion model to test the effects of pancreatic trypsin infusion on pain behaviors found that PAR-2-trypsin signaling mediated pancreatic pain independent of inflammation [29].

Our findings using both human pancreatic cancer tissues and a mouse orthotopic tumor model suggest that trypsin-PAR-2 signaling contributes to pancreatic cancer pain *in vivo*. Treatment strategies targeting PAR-2 or its downstream signaling molecules might effectively relieve and manage pancreatic cancer pain.

## MATERIALS AND METHODS

### Patients and tissue samples

The study protocol was approved by the Ethical Committee of the Eastern Hepatobiliary Surgery Hospital, Second Military Medical University, and was conducted from January 1, 2010 to January 31, 2013. Written informed consent was obtained from all patients. 22 patients who had mid abdominal pain and were scheduled for surgery with a tumor in the head of the pancreas were included in this study [30]. 22 patients diagnosed with common bile duct cancer and without abdominal pain were recruited as controls. Patient pain intensity was assessed using a visual analog scale (VAS, 0–10; 0: no pain, 10: worst possible pain) before surgery [31]. Pancreatic and common bile duct cancer diagnoses were confirmed by pathological examination after surgery. All patients had American Society of Anesthesiologists physical status I or II. Exclusion criteria included (1) age >75 or <18 years; (2) diabetes mellitus, or cardiovascular, respiratory, or renal diseases; (3) hepatic encephalopathy, psychiatric illnesses, or neuropathy; and (4) medications known to affect pain threshold. Pancreatic tissues were obtained after elective surgery for further analysis.

Supernatants from cultured human pancreatic cancer (PC supernatant) and normal pancreatic tissue (NC supernatant) samples were obtained as described previously [32]. Fresh specimens were cut into small pieces (20–50 mg) of approximately the same shape, rinsed in saline, and incubated immediately in Hank’s Balanced Salt Solution (HBSS) (100 mg specimens in 2 ml HBSS) at 37°C for 2 h. For all experiments, supernatant volumes were standardized to the weights of incubated biopsy samples and not to supernatant protein concentrations, as protein content was below the detectable threshold in biopsy supernatants. After incubation, supernatants were immediately centrifuged, concentrated 10-fold, frozen in liquid nitrogen, and stored at -80°C until use.

### Animals

All experiments were performed in accordance with the NIH Guide for the Care and Use of Laboratory Animals and the Ethical Issues of the IASP and were approved by the Second Military Medical University Committee on Animal Care. Male BALB/c nude mice aged 5–6 weeks and male Sprague-Dawley rats (200–250g) were purchased from the Slac Laboratory Animal Co., Ltd. (Shanghai, China). All animals used for this study were housed under a 12-h light/dark cycle in a pathogen-free area with *ad libitum* access to water and food.

### Immunofluorescence assays in human pancreatic specimens

Human pancreatic tissues were fixed in 4% paraformaldehyde overnight, then dehydrated for 48 h in 30% sucrose, embedded, and sectioned into 7-μm-thick slices. Anti-PAR-2 (1:2500, Abcam, Cambridge, MA) and the neuron marker, protein gene product-9.5 (PGP9.5) (1:1000, Abcam), were used for IF assays. All slides were observed and photographed under an Olympus microscope (IX-70 OLYMPUS, Tokyo, Japan).

### Trypsin levels and protease activity in human pancreatic specimens

Trypsin-like activity was measured using a continuous spectrophotometric enzymatic assay as previously described [33]. Activity was measured at 25°C following addition of the specimen supernatant (200 μl) to 3 ml of 0.25 mM BAEE substrate solution (Na-benzoyl-L-arginine ethyl ester (Sigma, St. Louis, MO) in 67 mM sodium phosphate buffer, pH 7.6). Absorbance at 253 nm was immediately recorded for approximately 5 min and ΔA253 nm/minute was obtained using the maximum linear rate. The result was standardized to the rate generated by known trypsin concentrations and normalized as described.

### DRG neuron primary cultures

Sprague-Dawley Rats were anesthetized via intraperitoneal injection of 80mg/kg sodium pentobarbital (Sigma) before being decapitated. DRG from all spinal levels were dissected, incubated at 37°C in enzymatic digestion solution for 25–30 min, and washed twice with Hanks solution. The enzymatic reaction was stopped via addition of Soybean trypsin inhibitor (1.25 mg/mL type II-S; Sigma). Ganglia were gently suspended using glass pipettes, transferred to poly-lysine coated slides, and allowed to stand for 2 h to settle the neurons on slides. Finally, slides were incubated in Dulbecco’s modified Eagle medium (DMEM; Biosource, Camarillo, CA) containing 10% fetal bovine serum (FBS) at 37°C in a humidified atmosphere with 3% CO_2_. Neurons were used within 2–12 h.

### RNA extraction and quantitative real-time PCR to detect SP and CGRP levels in cultured DRG neurons

Total RNA was extracted from treated sensory neurons using Trizol (Invitrogen, Grand Island, NY). First-strand cDNA was synthesized from 1 μg of total RNA using 200 units of Molony murine leukemia virus reverse transcriptase, 5 μM of random hexamers, and 1 mM of dNTP mix in a total reaction volume of 20 μL. cDNA was then diluted 10 times with dH_2_O and stored at −20°C. PCR was run in a total volume of 20 μL reaction mix containing TaqMan Universal PCR Master Mix and gene expression assay probes, using the Rotor Gene 3000 system (Corbett Research, Sydney, Australia). Gene expression probes used in this study were 4352340E, Rn01500392-m1, and Rn01511353-g1 for measuring β-actin, substance P precursor pre-protachykinin (PPT), and CGRP, respectively. Samples were run in duplicate. To normalize gene expression between different samples, mRNA levels were estimated as the ratio of gene X/β-actin. Data were expressed relative to the untreated sample.

### Enzyme-linked immunosorbent assay (ELISA)

Total protein was assayed for all sensory neuron culture supernatant samples. ELISA kits for SP and CGRP were used (Abcam) according to the manufacturer’s instructions, and microplates were read using a microplate reader (Molecular Devices, Sunnyvale, CA). Data were expressed relative to the normal sample.

### Mechanical hyperalgesia test

To assess mechanical hyperalgesia, animals were acclimated daily for 10 min/d for 3 d to the test environment, which was a plexiglass box on a metal grid surface. On test days, rats were allowed to acclimate for 5–10 min. The nociceptive stimulus, a single rigid filament attached to a hand-held transducer (electronic Von Frey anesthesiometer; IITC, Woodland Hills, CA) was applied perpendicularly to the medial surface of the hind paw with increasing force. The endpoint was taken as nocifensive paw withdrawal accompanied by head turning, biting and/or licking. As soon as this reaction occurred, the required pressure (indicated in grams) was considered the individual PWT value. Both hind limbs of each rat were tested in triplicate per time point. We also measured the required pressure for withdrawal responses to rigid filament application to the abdomen [34]. Any of the following behaviors on application of a hair was considered a withdrawal response: (a) sharp retraction of abdomen; (b) immediate licking or scratching of site of application; (c) jumping.

### Thermal hyperalgesia (Hargreaves) test

PWL was measured for both paws as previously described [35]. In brief, rats were placed under an inverted clear plastic chamber on a glass surface. After an adaptation period of 30 min, a radiant heat stimulus was applied to the plantar surface of each hind paw from underneath the glass floor (automatic plantar analgesia tester; Institute of Biomedical Engineering, China). A cutoff time of 25 sec was imposed to prevent tissue damage. Paws were alternated randomly to preclude order effects and PWL was determined as the mean of three measurements per paw.

### Ambulatory-evoked pain

Rats were placed in a large plastic observation box with a smooth floor. According to the extent of limb use during spontaneous ambulation, scores were characterized as follows: 0, normal use; 1, slight limp; 2, severe limp; or 3, complete lack of limb use. Testing was blinded with respect to groups [36].

### Hunching score

Hunching was measured as previously described from the first observation and continued weekly until euthanasia [37]. In briefly, mice were placed individually in the center of an open field arena (100 cm diameter, 30 cm high wall) with solid black walls and a black floor. Hunching behavior was scored from 0 to 4: 0, normal; 1, mild hunching; 2, severe hunching; 3, severe hunching with reduced movement; and 4, no movement. Observers were blinded with respect to groups, with two independent investigators confirming results.

### Pancreatic cancer model

The human pancreatic cancer cell line, SW1990, was purchased from the American Type Culture Collection. Cells were maintained in DMEM (Biosource) supplemented with 10% FBS, 100 units/ml penicillin, and 100 μg/mL streptomycin in a humidified incubator with 5% CO_2_ and 95% air at 37°C. Cells were released from plastic via 0.1% w/v trypsin, and then collected by centrifugation in 10 ml of medium for 3 min at 1200 rpm. The pellet was washed twice with 10 mL of DMEM, resuspended in 3 mL of DMEM, and then cells were counted using a hemocytometer and trypan blue solution. Cells were diluted to the final concentration for injection and kept on ice. For the sham group, SW1990 cells were diluted to final concentrations for injection and then boiled for 20 min.

Male BALB/c nude mice aged 5–6 weeks were maintained in a barrier facility on HEPA-filtered racks. For orthotopic tumor cell injection, mice were anesthetized with sodium pentobarbital (40 mg/kg) and a 0.5–1 cm incision was made in the left subcostal region. Tumor cells (5×10^6^) in 20 μL were injected into the body of the pancreas [38]. The pancreas was then returned to the peritoneal cavity, and the peritoneum and skin were closed with a 4.0 surgical suture.

### Electromyographic recording

VMR in pancreatic pain was previously described [12]. EMG recordings from the acromiotrapezius, a superficial dorsal skeletal muscle group, correlated best to the noxious stimulus in pancreas. A pair of electrodes (MYO/WIRE; A&E Medical Corporation, Farmingdale, NJ) was sutured into the acromiotrapezius muscle in the neck and externalized; the skin was sutured, and the externalized tubing and electrodes were covered with tape. Rats were allowed to recover from surgery. EMG recording was applied at 10:00 AM everyday after an adaptation period of 30 min. Three 10-min recordings were achieved with a 10-min interval period each day. The EMG response was amplified and filtered with 5-kHz low-pass and 10-Hz high-pass filters and digitized with BIOPAC software (Biopac Systems, Goleta, CA). From the rectified EMG, the area under the curve (AUC) was calculated using a computer program (Biopac Systems, Goleta, CA).

### Statistical analysis

Data analysis was performed using Origin 7.0 (OriginLab Corporation, Northampton, MA). All results are expressed as means ± SEM. All data were normally distributed. Between-group comparisons were performed using Student’s unpaired 2-tailed t test. Multiple comparisons within groups were performed using repeated measures one-way ANOVA, followed by Tukey’s procedure. The Mann-Whitney U test was used to compare nociceptive scores in animals. P<0.05 was considered significant.

## SUPPLEMENTARY MATERIALS FIGURE


