# Supplementary material for: Trypsin-protease activated receptor-2 signaling contributes to pancreatic cancer pain
Source: Oncotarget. 2017 Jun 27;8(37):61810–23. doi: 10.18632/oncotarget.18696 (PMC5617466; doi:10.18632/oncotarget.18696)
Supplement: Supplementary file 1 [file oncotarget-08-61810-s001.pdf]

Trypsin-protease activated receptor-2 signaling contributes to pancreatic cancer pain

SUPPLEMENTRY MATERIALS

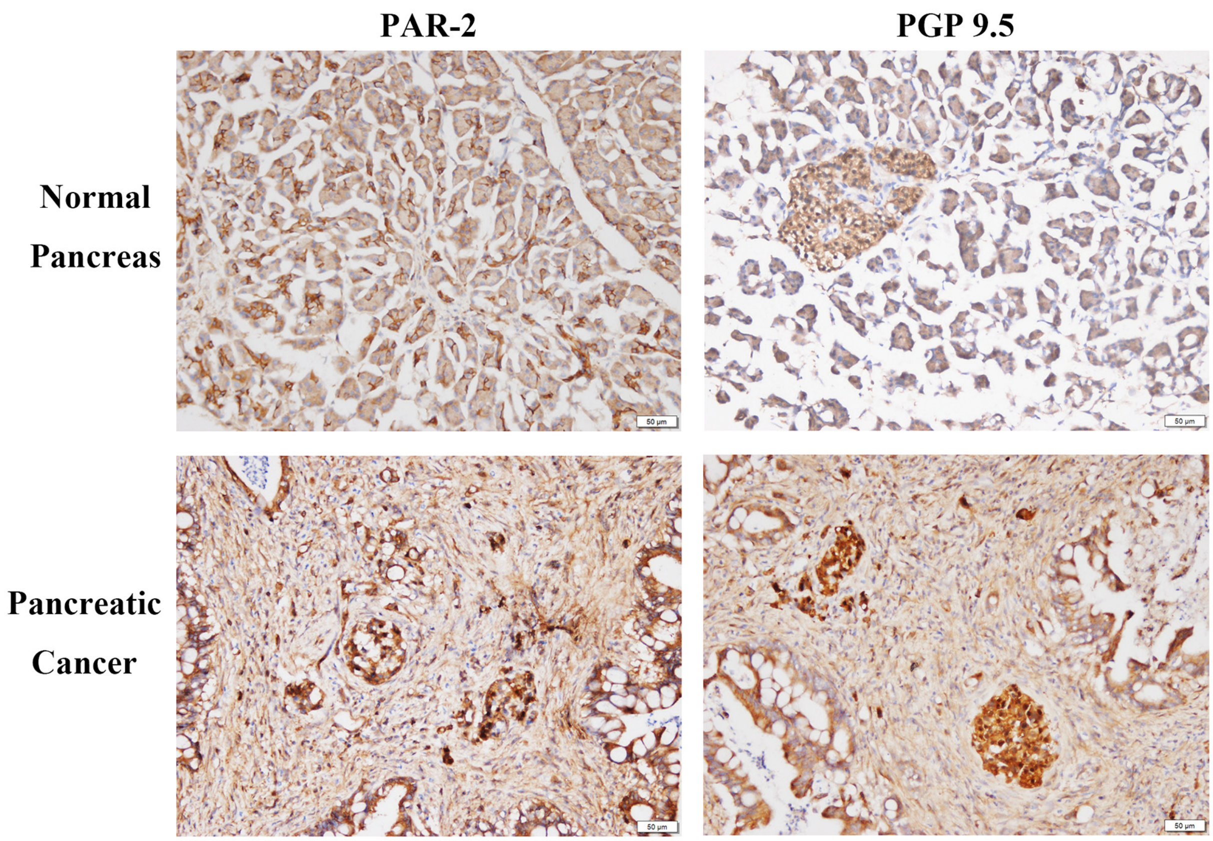

Supplementary Figure 1. Immunochemistry study for PGP 9.5 and PAR-2 expression in human pancreatic tissues. We also did immunochemistry study for PAR-2 and PGP 9.5 expression in human pancreatic normal and cancer tissues, and our statinning pattern of PGP9.5 immuno-active signals is the same as which had been reported as neurons in pancreas in previous study. Scale bar = 50μm.
